# Eighteen years of upland grassland carbon flux data: reference datasets, processing, and gap-filling procedure

**DOI:** 10.1038/s41597-023-02221-z

**Published:** 2023-05-23

**Authors:** Bruna R. Winck, Juliette M. G. Bloor, Katja Klumpp

**Affiliations:** Université Clermont Auvergne, INRAE, VetAgro Sup, UMR Ecosystème Prairial, 63000 Clermont-Ferrand, France

**Keywords:** Environmental impact, Projection and prediction

## Abstract

Plant-atmosphere exchange fluxes of CO_2_ measured with the Eddy covariance method are used extensively for the assessment of ecosystem carbon budgets worldwide. The present paper describes eddy flux measurements for a managed upland grassland in Central France studied over two decades (2003–2021). We present the site meteorological data for this measurement period, and we describe the pre-processing and post-processing approaches used to overcome issues of data gaps, commonly associated with long-term EC datasets. Recent progress in eddy flux technology and machine learning now paves the way to produce robust long-term datasets, based on normalised data processing techniques, but such reference datasets remain rare for grasslands. Here, we combined two gap-filling techniques, Marginal Distribution Sampling (short gaps) and Random Forest (long gaps), to complete two reference flux datasets at the half-hour and daily-scales respectively. The resulting datasets are valuable for assessing the response of grassland ecosystems to (past) climate change, but also for model evaluation and validation with respect to future global change research with the carbon-cycle community.

## Background & Summary

Long-term carbon (C) flux measurements are critical to assess both the patterns and drivers of ecosystem function over space and time. Eddy covariance (EC) measurements are a direct and instantaneous way to measure carbon fluxes and energy between atmosphere and surface. In recent years, networks of flux towers (EC measurements) have played a pivotal role in improving understanding of broad-scale carbon budgets and responses to abiotic and biotic factors both across and within contrasting ecosystems^1^. Although the installation of EC systems has increased worldwide (i.e., NEON, Ameriflux, AsiaFlux, ICOS), generating more available and reliable datasets based on standardised data-processing pipelines, the availability of long-term grassland flux datasets lags behind that of woody systems^2^. Long-term grassland flux studies hold great potential for identifying and understanding effective approaches to mitigate and adapt to global changes, including the provision of ecosystem services at a global scale.

Here, we describe 18-year datasets of greenhouse gas (GES) fluxes from an EC tower located in an upland permanent grassland site in the French Massif Central region, along with the methodology used for the pre- and post-processing of the data^3^. The production of accurate long-term eddy flux datasets relies on a suite of software and statistical tools for data pre- and post-processing^4^. Three general steps have a key effect on the quality of the final data in long-term eddy flux datasets: (i) raw-data pre-processing, (ii) time series discontinuity, that is, the number and length of gaps, and (iii) the gap-filling techniques (also called “imputation”). Data gaps in EC time series may be related to technical failures and/or changes in analyser technology, often non-randomly located across the EC time series, as well as to data quality checks (i.e., rejection of low-quality C fluxes^5,6^), which are typically randomly located in the time series^7^. Further, data measured in periods of low turbulence, which occurs mainly at nighttime, are rejected, thus generating more gaps^7,8^. Standard gap-filling methods based on Marginal Distribution Sampling (MDS^9^) are effective for short gaps^7^ because the missing value is replaced by the average of the response variable under similar weather conditions in a small-time window. However, recent studies show that MDS has low accuracy and high uncertainty when dealing with long gaps^10,11^. To overcome problems of long gaps in EC datasets, a variety of machine learning (ML) techniques (i.e., Random Forest and artificial neural networks) have been used to reconstruct long-term EC time series^10–12^. The application of ML techniques to flux data has the potential to provide robust gap-filling and requires few predictive variables to be measured continuously over long time periods^10,12,13^. Moreover, ML considers the temporal dependence and structure of the time series (i.e., trend and seasonality) and can deal with “noise” and complex interactions between variables^10^. In the present work, we therefore combined different statistical techniques to gap-fill data gaps of different origin and length in our EC time series, i.e., MDS and Random Forest techniques, generating two complete flux datasets (half-hourly and daily scale).

Our grassland study site is managed with low intensity cattle grazing typical for the region^14,15^, and the tower-based measurements include ecosystem-atmosphere turbulent fluxes of CO_2_ and H_2_O. The main products presented are: (1) half-hour data of C fluxes and energy with their respective quality flags and related meteorological variables (temperature, precipitation, radiation) from the onsite meteorological station; (2) gap-filled half-hourly NEE under three uStar threshold percentiles; (3) half-hourly C flux partitioning using night-time and daytime methods; and (4) gap-filled meteorological and C flux variables at the daily (diel) scale (daytime/night-time), accounting for long gaps^3^. To explore changes in C flux results as a function of pre- and post-processing techniques used in this paper, we also present a comparative analysis of parameterisation steps and C fluxes between the present analysis, and a previous shorter analysis of daily fluxes at the same site (2003–2011)^14^. Our datasets will be useful for exploring grassland ecosystem responses to environmental disturbances such as climate anomalies, the detection of possible early warning signals and tipping points, as well as providing a valuable resource for biogeochemical modelling and the prediction of grassland responses to future climate change.

## Methods

### Study site

**Fig. 1:**
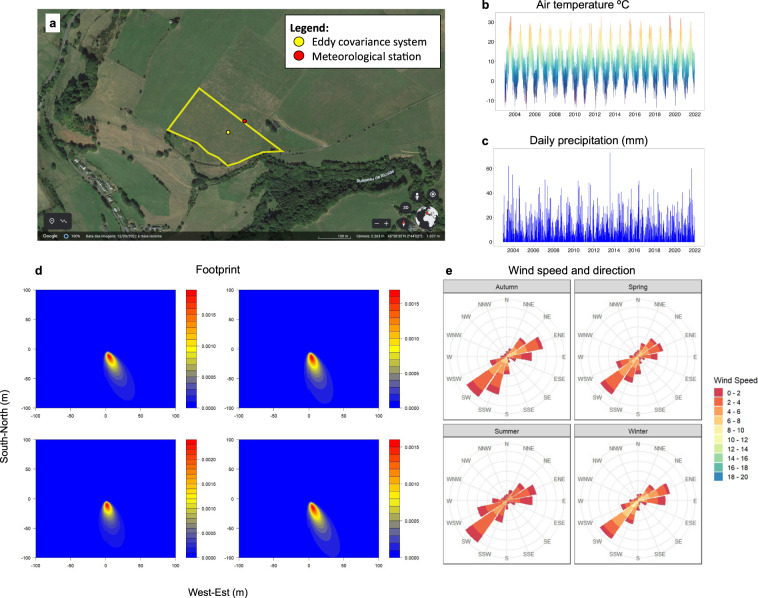
(**a**) Grassland management, (**b**) daily precipitation (mm), (**c**) temperature (°C), (**d**) seasonal CO_2_ footprint, and (**e**) seasonal wind rose at Laqueuille site, France during the study period.

The study site is located in an upland semi-natural grassland in the Auvergne region of France (1040 m asl; 45°38′N, 2°44′E) (Fig. 1) and has been under permanent grass cover since the 1950s. The local climate is classified as Cfb (Temperate oceanic climate) according to the Köppen classification; mean annual temperature and precipitation are 8.05 °C and 1073 mm, respectively (INRAe Climatik platform, 2022). The soil is an Andosol (20% clay, 53% silt and 27% sand) with carbon content ranging from 100 to 104.1 g kg^−1^and average bulk soil density of 0.87 g cm^−3^.

Since 2002, an experimental field (3.4 ha) has been managed by cattle grazing under low animal stocking rate (0.51 LSU ha^−1^ yr^−1^), with continuous grazing during the plant growing season (late April to late October). Vegetation is dominated by grasses including *Dactylis glomerata*, *Holcus mollis, Poa pratensis* and *Agrostis capillaris*. For full details on the experiment, see Allard *et al*. (2007) and Klumpp *et al*. (2011).

### Data processing and post-processing

**Fig. 2:**
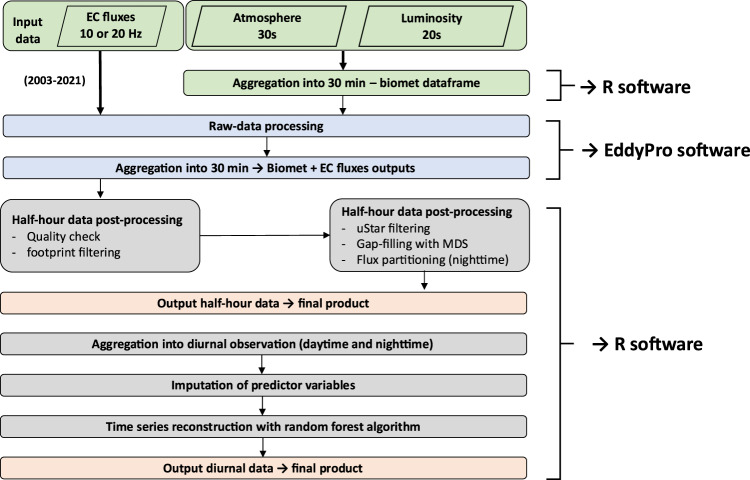
Workflow of pre- and post-processing step for half-hour and diurnal-daily data.

The workflow showing the steps of raw-data pre-processing and post-processing can be found in Fig. 2.

#### Eddy covariance and meteorological systems

Continuous measurements of surface-atmosphere exchanges of CO_2_ and H_2_O have been carried out in the extensively managed field since the start of the experiment (spring 2002). Flux measurements are done using an Eddy Covariance (EC) system installed at a height of 2 m (hereafter, “EC tower”). The tower is equipped with a high frequency sonic anemometer (Model Solent R3; Gill Instruments, Lymington, UK) to measure wind speed components (u, v, w) and an open-path analyser to measure CO_2_ and H_2_O (Model LI-7500; LI-Cor Inc., Lincoln, NE, USA). Data is recorded at 10 to 20 Hz and recorded on a computer and datalogger^14,15^.

The site is equipped with a meteorological station that provides high frequency measure of atmospheric (Tair: air temperature, RH: relative humidity, PA: atmospheric pressure, P: total precipitation, ws: wind speed, wd: wind direction) and solar radiation (PPFD: photosynthesis active radiation, Rg: global radiation, Rn: net radiation). The frequency for atmospheric and solar radiation is 30 and 20 seconds, respectively.

#### Flux data processing and post-processing

**Fig. 3:**
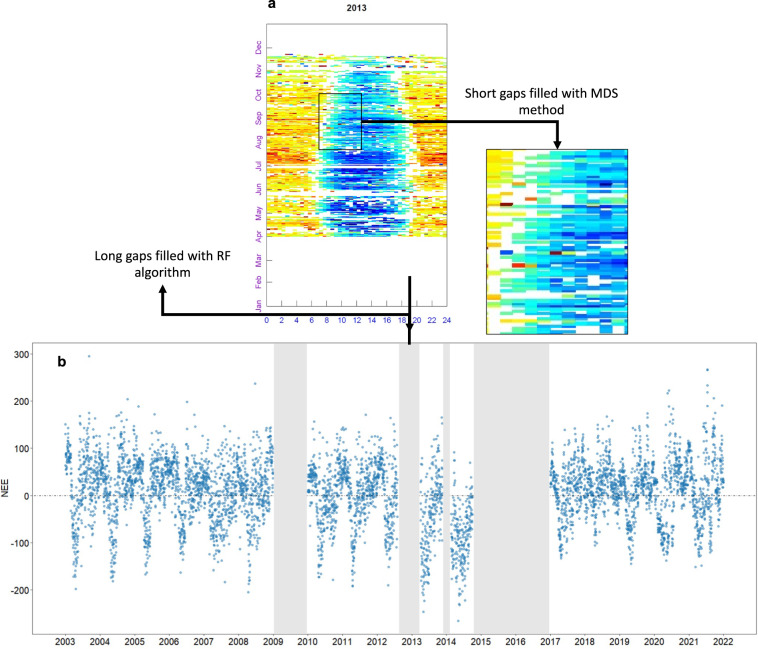
Gaps in net ecosystem exchange (NEE) data at the grassland site, Laqueuille, France. (**a**) fingerprint showing gaps in half-hour NEE data; (**b**) time series of daily NEE data showing long gaps.

Raw-data (10 Hz until 2016 and 20 Hz onwards) from the EC tower and meteorological station were pre-processed with EddyPro® software (Li-COR, version 7.0.9) following the processing steps and methods^16–27^ presented in Supplementary Table 1 and Table 1. Processed data was converted into half-hourly flux data and post-processing was performed following international recommendations of FLUXNET^2^ using R Studio Software. In brief, post-processing steps included: (i) data filtering of low-quality values of NEE, (ii) filtering of values outside the footprint area^20^, (iii) filtering of values under low friction velocity (uStar), (iv) gap-filling of missing values using the MDS method^11^ for half-hour data (shorter gaps), (v) partitioning net ecosystem exchange (NEE) into ecosystem respiration (R_eco_) and gross primary productivity (GPP), based on the nighttime and daytime algorithms^9,28^, (vi) gap-filling of missing values using RF algorithms for daily data (long gaps)^2,10^. Short gaps are random gaps often produced during data quality check that were distributed throughout the EC time. On the other hand, long gaps are non-random gaps that are mainly related to instrumental failures or changes, and they located in specific points across the EC time series. For instance, in our EC time series we identified four long gaps (Fig. 3), the largest gap being a sequence of 26 months, from October 2014 to December 2016. Post-processing steps are described in detail below.

**Table 1:** Comparison of post-processing steps applied on half-hour and diurnal-daily data in the present study and that of Klumpp *et al*. (2011).

Post-processing steps	Klumpp *et al*. (2011)	This dataset^a^
Remove values beyond the physical boundaries	−50 to 50 for CO_2_, −250 to 1000 for LE, −250 to 1100 for H	*FREddyPro::cleanVar ‡* (−50 to 50 for CO_2_, −250 to 1000 for LE, −250 to 1100 for H)
Despiking	Removed at pre-processing (see Supplementary Table 1)	Removed all values flagged as 1 (not passed) during statistical test performed during the pre-processing in EddyPro (Mauder and Foken, 2004)
Remove Spectra and co-spectra	Removed all values flagged as 2	*FREddyPro::qcClean* (removed all values flagged as 2)
Remove Values based on standard deviation of means	See Supplementary Table 1 for detailed conditions	*FREddyPro::sdClean* (removed all values higher than 3 standard deviation)
Remove values based on outliers	See Supplementary Table 1 for detailed conditions	*FREddyPro::removeOutliers* (removed all values below the 25th percentile or above the 75th percentile)
Remove values lower than a given u* threshold	Fix u* = *0.8*	*REddyProc::sEstimateUstarScenarios ‡*, using seasonal threshold (Wutzler *et al*., 2018)
Remove values beyond the field boundaries (footprint check)	Not taken into account	Removed all values for which the x_peak was beyond the footprint
Gap-filling half-hour data (short-gaps)	*REddyProc::sMDSGapFillUStarScens*	*REddyProc::sMDSGapFillUStarScens*
Flux partitioning into GPP and R_eco_ (nighttime method)	*REddyProc::sMRFluxPartitionUStarScens*	*REddyProc::sMRFluxPartitionUStarScens*
Flux partitioning into GPP and R_eco_ (daytime method)	Not taken into account	*REddyProc:: sGLFluxPartitionUStarScens*
Gap-filling daily data (long-gaps)	Not taken into account	*RF algorithm (several R packages)*

^a^*package::function* in R software (when applied).

#### Data quality check

The quality check procedure for the half-hour data was performed in six steps (Table 1) using the R packages “*FreddyPro*”(https://github.com/cran/FREddyPro) and “*REddyProc*”^8^:

- Physical boundaries: Data were rejected when beyond the physical boundaries considered for this experimental site: CO_2_ (−50 to 50 μmol CO_2_ sec^−1^ m^2^), LE (−250 to 1000 W m^−2^), H (−250 to 1000 W m^−2^), and VPD (0 to 50 Pa).
- Quality control (QC) flags: EddyPro software assigns QC flags based on the combination of both steady-state turbulence and well-developed turbulence tests, where the flag “0” represents high-quality fluxes, “1” intermediate-quality fluxes, and “2” represents low-quality fluxes^29,30^. Following the recommendation of Vitale *et al*. (2020), we rejected all low-quality fluxes, flagged as “2”.
- Raw data statistical screening: Based on nine statistical tests to check unusual behaviours in the time series, EddyPro software assigns two hard flags for each half-hourly data, where “0” represents “passed” and “1” represents “failed”. Data with a hard flag of 1 for the spike test were rejected. The quality check results related to all other statistical screening procedures (Supplementary Table 1) are presented in the dataset.
- Standard deviation and outliers: We rejected data with values greater than 3 standard deviations from the mean positive and negative values of the complete EC time series (i.e., outliers from the interquartile range with 75th and 25th percentiles).
- Footprint: Data were filtered with respect to field margins to minimize the risk of fluxes from outside the field. We rejected values where the distance between the tower and the peak was greater than that of the fetch, so that only values in the target area remained.
- uStar: Data were filtered for insufficient atmospheric turbulence (i.e., mostly at night) using multiple uStar thresholds (0.05, 0.5, 0.95 quantiles) during the year to account for seasonality in vegetation and climate classes (air temperature and precipitation). The uStar thresholds were estimated using the bootstrapping method^31^ (n = 1000 resamples).

The percentage of missing values before and after data cleaning by day and diel period is given in the XLSX file “**FR_Lq2_EXTENSIF_Li_7500_CR3000_2003_2021_gaps**.**xlsx”**.

#### Gap-filling of short gap periods and C flux partitioning

Following data quality checks, short gaps in NEE were imputed using Marginal distribution sampling-MDS^8^ as recommended by FLUXNET^2^, using the R package “*REddyProc*”^8^. The MDS combines two gap-filling techniques: the “look-up table” and the “mean diurnal course”. In essence, the MDS technique creates look-up tables which seek similar meteorological conditions (global radiation Rg, air temperature Tair, and vapor pressure deficit VPD) under different window sizes that are physically and temporally similar to the missing data and imputes them using the average values. The meteorological conditions are considered similar when they do not vary more than 50 W m^−2^, 2.5 °C, and 5, hPa respectively. When all the meteorological variables are available in a 7-day window, the gap is filled by the mean value. When MDS fails to find similar meteorological data, the search continues and considers only the presence of Rg, and the gap is filled with the mean value in a 7-day window. When no appropriate similar conditions are available, the gap is filled using diurnal curve courses, which replace the gaps with the mean value for the exact time of day of the adjacent days^32^. If the gap still exists after these steps, the same procedure is carried out using progressively larger time windows^31^.

After the gap-filling procedure, different gap-filled NEE (*NEE_f*) are generated, including their uncertainties (*_fsd*), distinguished by a suffix with the quantile (_05, _50, and _95). The final gap-filled NEE were partitioned into GPP (*GPP_f*) and R_eco_ based on standard night-time and daytime algorithms^9,28^,also distinguished by a suffix with the quantile (_05, _50, and _95). The night-time method uses night-time NEE to fit a respiration model based on the relationships between NEE and air temperature. GPP is inferred by extrapolating R_eco_ to daytime temperature and by subtracting the latter term from NEE. The daytime algorithm uses daytime and night-time NEE to calibrate a model based on light-response curves and VPD to predict GPP, and the relationship between temperature and respiration to predict R_eco_, as with the night-time method.

#### Uncertainty in gap-filling of C flux and uStar threshold

The most significant sources of uncertainties in the post-processing of half-hour data occur when estimating the uStar threshold and the gap-filling procedure. During the gap-filling procedure, searching for similar conditions attempts to keep the window size as small as possible. However, the more the variables are missing, the larger the time window. As a result, this increases the uncertainty in gap-filling, which is flagged (*_F_MDS_QC*) as follows: 0 (measured); 1 (high confidence imputation); 2 (medium confidence imputation); and 3 (low confidence imputation). To visualise the uncertainty associated with the uStar filtering, we computed uStar thresholds using a large sequence of quantiles ranging from 0.025 to 0.975 (nSample = 1000 L, length.out = 39). The greater the difference between the extreme the greater can be the uncertainty introduced by uStar filtering. The time sequence with low data quality or the absence of measurements were excluded from this analysis. Uncertainties associated with the daily sum of NEE were calculated using the standard deviation of the observations, considering the autocorrelation between the observations^33^. More detailed information regarding uncertainty analysis in aggregated NEE can be found at the following website: https://cran.r-project.org/web/packages/REddyProc/vignettes/aggUncertainty.html.

**Fig. 4:**
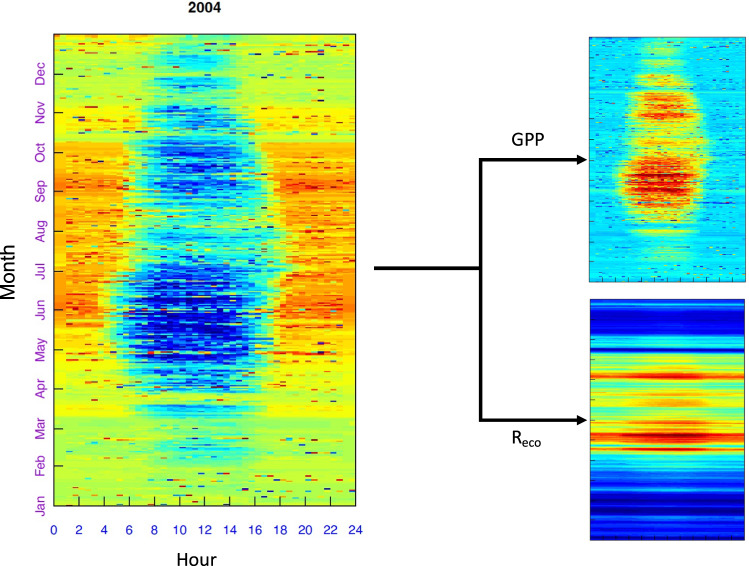
Example summary fingerprint plots of net ecosystem exchange (NEE), gross primary productivity (GPP), and ecosystem respiration (R_eco_) in 2004 after MDS gap-filling showing diurnal and seasonal C fluxes at the study site.

#### Gap-filling of long-term gaps and model uncertainty

Long gaps in C fluxes were filled using the random forest (RF) algorithm^34^ and a set of R packages (*parsnip*^35^*, recipes*^36^*, ranger*^37^*, rsample*^38^*, tune*^39^*, workflows*^40^). RF is a machine learning algorithm that uses an ensemble-learning method based on regression trees; predictions from multiple decision trees are aggregated to generate more accurate predictions than a single model. Use of RF is robust in the presence of noise and in detecting complex relationships between variables, but its performance depends on the tuning of its hyperparameters, the number of features, and the dataset size. Typically, the more the training data are increased, the greater the model accuracy becomes, reducing overfitting. For time series, a complete sequence of data should be large enough to detect patterns such as trend and seasonality. Given that RF requires high computation performance and that C fluxes have different patterns with respect to time-of-day, we downscaled our data into diel observations per day (daytime/night-time). Daytime was defined by using the R function “*solartime::computeIsDayByLocation*”^41^. Detailed description of the variables for RF training is described in Table 2. Overall, following steps were performed to predict and impute long-gap periods:

**Table 2:** List of predictor and response variables used in the random forest models.

Label	Description	Statistical aggregation	Variable Type
***Response variables***			
NEE	Net ecosystem exchange	sum	continuous
GPP	Gross primary productivity	sum	continuous
R_eco_	Ecosystem respiration	sum	continuous
***Predictive variables***			
Date^a^	Split into several time series signature features	#	Category -> dummy
Period	Daytime/nighttime	#	Category -> dummy
Tair	Air temperature	mean	continuous
Tmin	Air temperature	minimum	continuous
Tmax	Air temperature	maximum	continuous
VPD	Ambient water vapour pressure deficit	mean	continuous
VPDmin	Ambient water vapour pressure deficit	minimum	continuous
VPDmax	Ambient water vapour pressure deficit	maximum	continuous
PPFD	Photosynthetically active radiation	sum	continuous
RH	Relative moisture	mean	continuous
RHmin	Relative moisture	minimum	continuous
RHmax	Relative moisture	maximum	continuous
P	Total precipitation	sum	continuous
Rg	Global radiation	sum	continuous
Rn	Net radiation	sum	continuous
Pa	Air pressure	mean	continuous
LE	Latent heat flux	sum	continuous
H	Sensible heat flux	sum	continuous
Ustar	Friction velocity	mean	continuous
ws	Wind speed	mean	continuous
wd	Wind direction	mean	continuous
anom_p	Precipitation variability	percentage^b^	continuous
anom_t	Temperature variability	percentage^b^	continuous

^a^During RF model building, the Date variable was used to create time series signature features (i.e., day of the year, day of the month, weekday). These variables are used to detect temporal patterns in the input dataset.

^b^Variations in percentage in relation to climatological normal calculated over a 30-year period.

#### Response variables

- We used the daily sum of NEE (NEE_U50_f), R_eco_ (Reco_U50), and GPP (GPP_U50_f) as response variables in the RF models.

#### Predictor variables

- The mean, minimum and maximum of variables describing meteorological conditions (uStar, Tair, P, RH, VPD, ws, and wd) and solar radiation (Rg, Rn, and PPFD) were inserted as predictor variables in the RF models. The minimum and maximum values are thought to capture the daily variation of the predictor variable. In view of the strong and bidirectional relationship between energy fluxes, often related to evapotranspiration processes, and C fluxes^35^, LE and H were also inserted as predictors.
- Anomalies of temperature (t_anom) and precipitation (p_anom) were included as additional predictors. Both variables were calculated as the difference of the observed value in relation to the climate “norm” of the reference month. The climate “norm” was calculated over a 30-year period using data from Laqueuille meteorological station (INRAe Climatik platform, 2022, https://internet.inra.fr/climatik), in line with recommendations by the World Meteorological Organization^42^.
- Because RF algorithms do not deal with missing values in predictors, those variables were previously gap-filled using the R function “*missForest::missForest*”^43^ with 200 trees and 5 interactions. Since the out-of-bag (OOB) error was around 0.03, which indicate high performance of the gap-filling method, we use these imputed meteorological variables in the next steps of the RF analysis.

#### Model training

- The EC time series after MDS gap-filling was 100% complete between 2003 and 2008 for all response variables. Therefore, we used this sequence to generate the training and testing datasets. The time sequence from 2003 to 2007, corresponding to 70% of the data, was used to train the RF models and predict NEE, R_eco_, and GPP in 2008. The testing dataset (2008), corresponding to 30% of the data, was subsequently used to validate the RF models.
- RF models were built using the R functions “*recipe*”, “*bake*”, and “*juice*” from the “*recipe”*^36^ package. During RF model building, we insert all the aforementioned predictors, as well as the time series signatures using the R function “*timetk::step_timeseries_signature”*^44^. Time series signatures use the “Date” column to generate a set of time-based features (i.e., day of the year and the month, week of the year, day of the wee, month, quarter) that define when each observation occurred. These signatures can capture common seasonal and trend patterns of a given time series. Continuous variables were normalised to have a data deviation of one and a mean of zero, whereas all the categorical variables, including time series signatures, were converted into dummy variables. While data normalization improves model prediction by reducing the strong difference between the predictors, dummy transformation reduces model complexity, the computation time, and the bias related to the number of levels in each category.
- The models were trained using the R function “*parsnip::rand_forest”*^35^ with 500 decision trees, which is above the value at which the out-of-bag error stabilized, and tunned “*mtry*”^36^. Computational engine and prediction outcome mode were set as “*ranger*” and “*regression*”, respectively.
- During the model training, we checked the importance of all predictors and we excluded those of low importance in a step-wise manner. This procedure was repeated until root mean squared prediction error (RMSE) was found to increase and R^2^ to decrease. When this happened, the last variable to be removed was re-inserted in the final model, and this was used in the validation step.

#### Model validation

- The validation of the models was carried out by predicting the entire year of 2008 and comparing it with the testing dataset. The models with the highest (R^2^) between the predicted and observed values were chosen for gap-filling of missing values in NEE, GPP, and R_eco_.
- To ensure high predictive capacity and lower uncertainty, each model was run 50 times. The average of the predicted values was used both in validation and in imputation, as well as to calculate the standard deviation (SD) of the coefficient of determination (R^2^), root mean squared prediction error (RMSE), and mean absolute error (MAE).
- As a further check of the validity of our RF models for the gap-filling procedure and the representativity of the climate for the years used in training step, we used 2004–2008 as an alternative training dataset to predict 2003 (an atypical year).

#### Sensibility of RF models to gap length and timing

- We evaluated the sensitivity of the RF models to gap length and location by generating testing datasets based on 2008; the complete dataset was altered to generate varying degrees of missing values (4, 14, 28, 41, 55, 69, 82, and 100%) starting from the 1^st^ day of the year. Artificial gap sequences were imputed using the trained RF models (2003–2007) described above. To test the sensibility to timing of gaps (gap location), we investigated the sensitivity of our RF models to a gap of constant length (2 months), positioned at different locations in the 2008 time series according to the seasons. The performance of the gap-filling procedure for each gap scenario was evaluated by analysing the final R^2^ and RMSE (same methodologies as above). The slope of the linear models between predicted and observed values was also used as a metric to evaluate the model sensitivity to gap length or location.

## Data Records

The long-term datasets (2003–2021) are distributed in files (CSV format, UTF-8 comma delimited) separated by temporal aggregation, e.g., half-hourly (HH suffixes) and daily split (daytime/night-time period, DD-DN suffixes). Each file is accompanied by its respective metadata in XLSX format, containing the full list of variables, the measurement units, and the variable description. The half-hour dataset is a complete dataset generated by the pre- and post-processing in EddyPro and REddyProc, respectively. This dataset contains 258 variables, including the original (_original suffixes) and gapfilled (_f suffixes) values for Rg, VPD, Tair, NEE, R_eco_, and GPP using the MDS technique. The daily dataset contains 31 variables aggregated from the half-hour dataset (_RF suffixes for gapfilled data and _original suffixes for non-gapfilled data) into daytime and nighttime period. We provide XLXS files describing the site and flux tower system, the animal stocking rate, and the number and percentage of gaps before and after the data quality check procedure. Finally, we provide a ZIP file with an example of EddyPro processing where all configuration steps can be checked. The prefix of the file names **“**FR_Lq2_EXTENSIF_Li_7500_CR3000_2003_2021_*” provides the follow information: country (FR = France), site (Lq2 = Laqueuille, ICOS code), grassland management (EXTENSIF = Extensive management), Li-Cor sensor (Li-7500 open-path), datalogger model (CR3000 Micrologger®), and the beginning and end of the time series. Details on the files names and their content are given in Table 3. All files are available for download as a single ZIP file through the public repository Dataverse INRAe^3^.

**Table 3:** List of dataset and contents. Country (FR = France), site (Lq2 = Laqueuille, ICOS code), grassland management (EXTENSIF = Extensive management), Li-Cor sensor (Li-7500 open-path), datalogger model (CR3000 Micrologger®), and the beginning and end of the time series.

Files names	File contents
FR-Lq2_EXTENSIF_Li_7500_CR3000_2003_2021_Site_Description.xlsx	Site description
FR-Lq2_EXTENSIF_Li_7500_CR3000_2003_2021_animal_stocking_rate.xlsx	Daily grassland management
FR_Lq2_EXTENSIF_Li_7500_CR3000_2003_2021_HH.csv	Output from eddypro and Reddyproc after processing and post-processing, meteorological data.
FR-Lq2_EXTENSIF_Li_7500_CR3000_2003_2021_HH_metadata.xlsx	List of variables and units, description of the variables
FR-Lq2_EXTENSIF_Li_7500_CR3000_2003_2021_HH_gaps.xlsx	Number and percentage of gaps before and after data quality check
FR_Lq2_EXTENSIF_Li_7500_CR3000_2003_2021_NA_QC_Table-S1.csv	Number and percentage of missing values (NA) before and after quality check
FR_Lq2_EXTENSIF_Li_7500_CR3000_2003_2021_DD-DN.csv	Aggregated data at diel resolution split into daytime-nighttime period, meteorological variables imputed with “*missforest”* package and outcome with random forest
FR-Lq2_EXTENSIF_Li_7500_CR3000_2003_2021_DD-DN_metadata.xlsx	List of variables and units, description of the variables
FR_Lq2_EXTENSIF_Li_7500_CR3000_2003_2021_Models_RF.csv	RMSE, R^2^, and MAE of the 50 random forest models for each response variable
FR_Lq2_EXTENSIF_Li_7500_CR3000_time_series_signature_Table-S2.xlsx	Description of time series signature used in RF models
FR_Lq2_EXTENSIF_Li_7500_CR3000_2003_2021_postprocessing - MDS.Rmd	R script of half-hour data post-processing
FR_Lq2_EXTENSIF_Li_7500_CR3000_2003_2021_postprocessing - RF.Rmd	R script to generate DD-DN datasets and random forest model performed using the DD-DN
FR_Lq2_EXTENSIF_Li_7500_CR3000.eddypro	Raw-data processing of 2021
FR_Lq2_EXTENSIF_Li_7500_CR3000.metadata	Metadata of raw-data processing in EddyPro of 2021
FR_Lq2_EXTENSIF_Li_7500_CR3000_2003_2021_planar_fit.txt	Input for raw-data processing in EddyPro of 2021
FR_Lq2_EXTENSIF_Li_7500_CR3000_2003_2021_time_lag.txt	Input for raw-data processing in EddyPro of 2021

HH: half-hour data, DD: daily data; DN: daily data split into daytime and nighttime, RF: random forest, MDS: Marginal distribution sampling, NA: missing values.

## Technical Validation

**Fig. 5:**
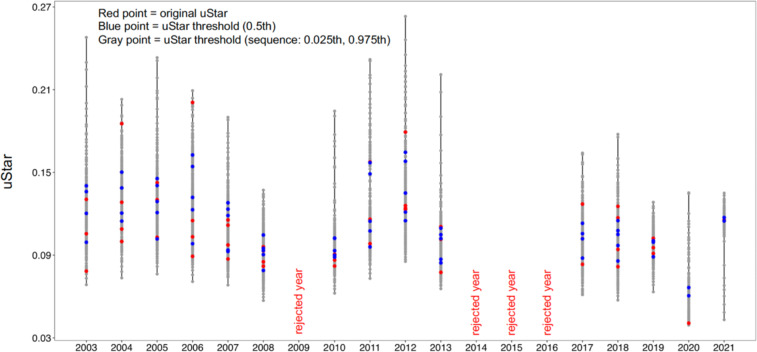
Ustar threshold for each year. Red point represents original Ustar by season, blue point the uStar threshold 0.5^th^, and grey points the uStar sequence ranging from 0.025^th^ to 0.975^th^ percentiles.

**Fig. 6:**
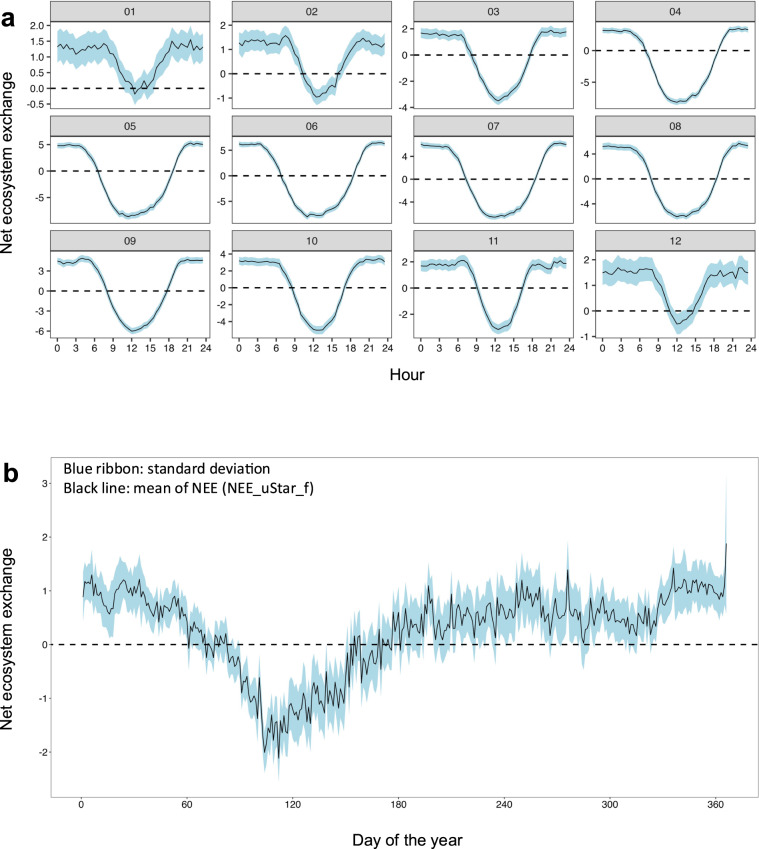
Uncertainties in aggregate net ecosystem exchange (NEE) an extensively-managed grassland, Laqueuille, France. (**a**) Hourly aggregation (black line) for each month and standard deviation (blue ribbon); (**b**) Daily aggregation (black line) and standard deviation (blue ribbon).

To ensure robust and high-quality flux of our results after the pre-processing using EddyPro, the output of the half-hour C-fluxes were visually checked using fingerprint plots. A typical fingerprint plot presents negative NEE (photosynthesis) values during daytime in summer and spring and positive NEE values (respiration) during at nighttime and in winter and autumn (Fig. 4). When the fingerprints were not as expected, suggesting low data quality or instrumental failures, the sequence was rejected from the time series and imputed using RF models. We also examined the uncertainties associated with the estimation of uStar thresholds (Fig. 5). The more dispersed are the uStar values, the greater their uncertainty. Figure 6 shows the mean diurnal and annual cycle of the NEE and the respective uncertainties. Uncertainty is higher in the colder months of the year (December-February) and during nighttime, possibly associated with the greater flux magnitude.

**Fig. 7:**
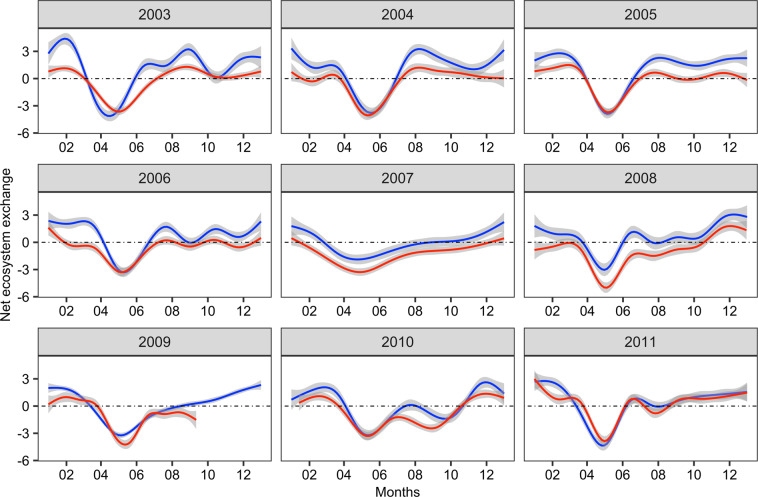
Daily mean of net ecosystem exchange (NEE) from 2003 to 2011 in an extensively-managed grassland, Laqueuille, France. Blue lines are reprocessed, and gapfilled raw-data performed in this study and red lines are the results from Klumpp *et al*. (2011).

Changes in NEE related to pre-processing and data filtering (i.e., missing values allowance, uStar, footprint) were assessed with respect to the choices made in a previous work using a subset of the same EC raw-data^16^. The pre-processing of the current dataset generated similar patterns of C flux over time to those generated by the raw-data pre-processing in a previous study^16^. However, our outputs were significantly higher at several moments along the EC time series between 2003–2011 (Fig. 7). Although raw data from Klumpp *et al*. (2011) was pre-processed using the EdiRe (no longer available) to estimate C flux, and here pre-processed with EddyPro, a previous work has shown that there is an agreement between both software when the pre-processing steps are similar^38^. Thus, we assume that observed differences between the C fluxes are likely due to the parametrization choices made during pre- and post- data processing (Supplementary Table 1 and Table 1). Some steps of data processing may have been critical in this difference. For instance, during the raw-data pre-processing, we applied a planar fit for tilt correction, while Klumpp *et al*. (2011) used double rotation. Likewise, algorithms used in spectral analyses, dropouts in the registration of raw data in 20 Hz compared to initial 10 Hz, as well as performances in low and high path filtering have been improved since the EdiRe software, providing slightly modified C flux estimations^39^. Finally, unlike Klumpp *et al*. (2011) who applied an annually fixed uStar thresholds (u* ~ 0.8) to filter the data under low friction velocity, we applied seasonal uStar thresholds that was estimated using nighttime NEE measurements and bootstrap procedure. Indeed, we found that sliding thresholds minimized the risk of excluding realistic and high-quality data which could lead to C-flux underestimation.

**Fig. 8:**
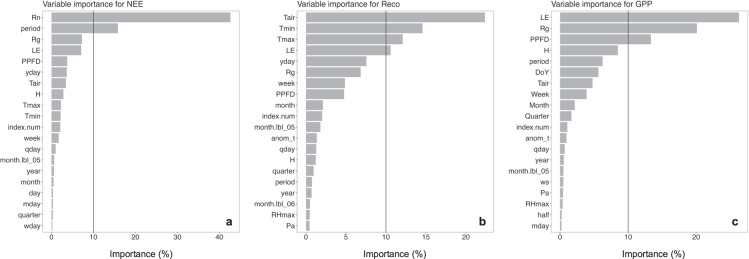
Final predictor variables considered each random forest model to predict NEE, R_eco_, and GPP.

The relative importance of the predictors used in RF models (training: 2003–2007, testing: 2008) for each response variable is given in Fig. 8. Our analysis revealed that the daily NEE, GPP, and R_eco_ values could be estimated by basic meteorological and radiation variables (Tair, Tmin, Tmax, Rg, Rn, PPFD), but also by energy fluxes (LE and H) and the time series signature. Meteorological variables can control C fluxes in different ways, either by affecting CO_2_ detection by the analyzer, or by affecting the ecosystem *per se*. For instance, the detection of CO_2_ by the analyzer can be reduced under low friction velocity, resulting in underestimated fluxes. Likewise, by influencing the performance of autotrophic organisms, mainly of plants, meteorological variables can alter the balance between respiration and photosynthesis, mainly under high climatic amplitude. On the other hand, the effect of LE and H on C fluxes seems to be mediated by their effect on water fluxes (evapotranspiration) and consequently stomatal closure of the plants. This physiological change can also alter the balance between respiration and photosynthesis in the ecosystem^45^.

**Fig. 9:**
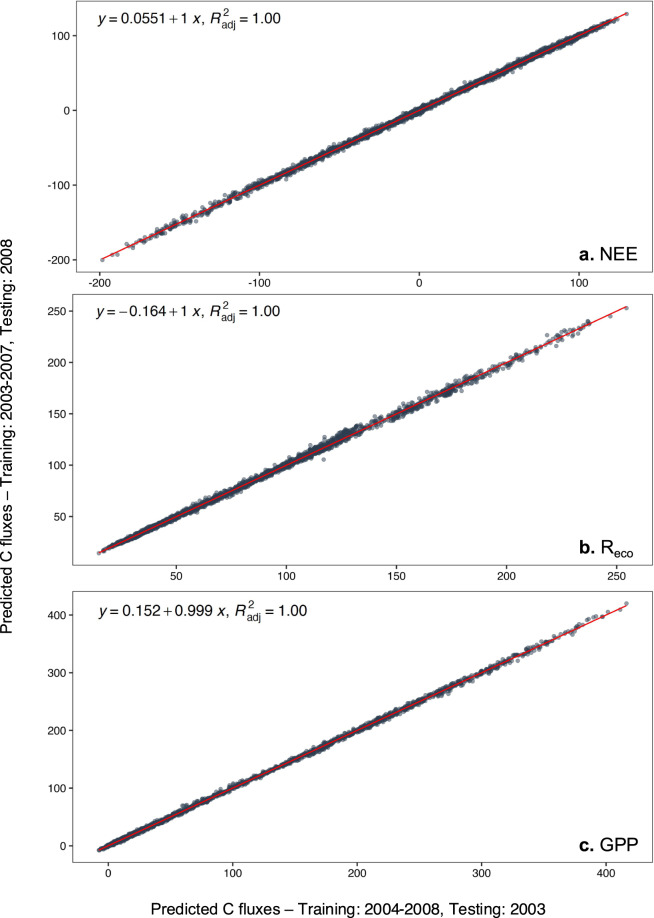
Linear model regressions between predicted values of NEE, R_eco_, and GPP using random forest algorithms trained with 2004–2008 (predicting 2003) and with 2003–2007 (predicting 2008).

**Fig. 10:**
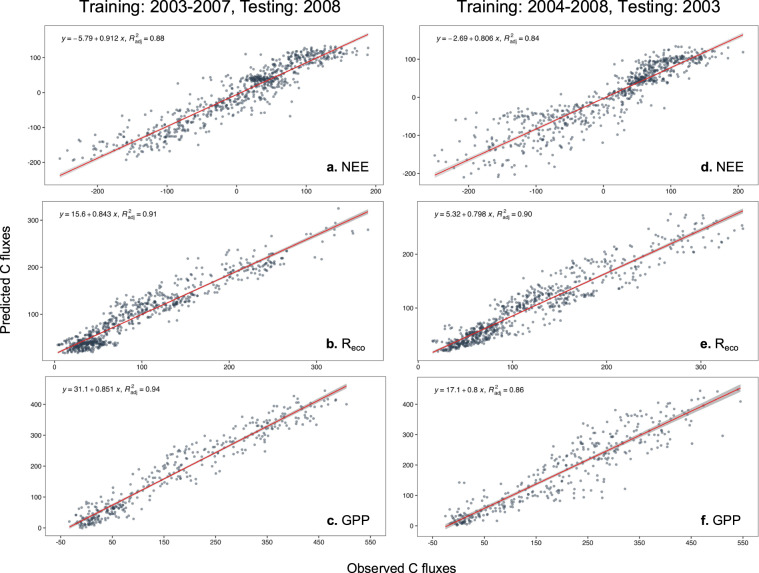
Linear model regressions between observed and predicted values of NEE, R_eco_, and GPP using random forest algorithm trained with 2004–2008 (predicting 2003) and 2003–2007 (predicting 2008) for the grassland study site.

Validation of the RF models using alternative training and testing datasets (either “training: 2004–2008, testing: 2003” or “training: 2003–2007, testing: 2008”) indicated that the two models resulted in very similar C flux output (Fig. 9). When predicting 2008, the cross-validation between predicted and observed values had R^2^ values > 0.85 in all cases, and slopes were 0.91, 0.84, and 0.85 for NEE, R_eco_, and GPP respectively (Fig. 10a–c). The prediction of 2003 (training set 2004–2008) also had R^2^ values > 0.84 for all flux variables but showed marginally-lower slopes values for NEE (0.81), R_eco_ (0.80), and GPP (0.80) (Fig. 10d,e). Overall, high R^2^ indicates that the RF models are not overfitting, whereas low slope values indicate low discrepancy of the fit between the observed and predicted values.

Sensitivity analysis of the RF models indicated no decrease in gap-filling reliability with respect to gap length in the 2008 test series (Table 4). Instead, the RF models actually improved their predictive capacity with increasing gap size, with a reduction of the intercept and slope values, and increases in R^2^. The improvement of the model performance for large gaps may be due to the insertion of time series signature features in the RF models, which better capture the seasonality and trends in the EC time series. Reliability of gap-filling tended to be lower for gaps during the winter period based on comparisons of R^2^ and slopes of when fitting observed and predicted C fluxes (Table 5), but the magnitude of change was not significant. These results confirm that the models were able to predict and fill gaps at different times of the year.

**Table 4:** Linear model metrics comparing observed and predicted C fluxes across a sequence of gap length (%).

Gap length	slope	R^2^	RMSE
***NEE***			
4%	0.80	0.91	10.65
14%	0.74	0.92	9.37
28%	0.81	0.96	8.95
41%	0.91	0.98	10.73
55%	0.95	0.99	11.41
69%	0.95	0.99	11.30
82%	0.96	0.99	11.00
100%	0.96	0.99	10.80
***R***_***eco***_			
4%	0.86	0.98	2.64
14%	0.85	0.96	2.30
28%	0.86	0.94	4.09
41%	0.94	0.99	5.73
55%	0.95	0.99	6.77
69%	0.95	0.99	6.86
82%	0.95	0.99	6.62
100%	0.95	0.99	6.22
***GPP***			
4%	0.78	0.95	8.58
14%	0.70	0.93	8.92
28%	0.79	0.96	9.45
41%	0.94	0.99	11.80
55%	0.96	0.99	11.88
69%	0.96	0.99	11.64
82%	0.97	0.99	11.51
100%	0.97	0.99	11.24

Slope of regression model, R^2^: coefficient of determination, RMSE: root mean square error. Random Forest training using the data range from 2003 to 2007, testing using 2008.

**Table 5:** Linear model metrics comparing observed and predicted C fluxes in different gap position (seasons).

Gap position	slope	R^2^	RMSE
***NEE***			
Winter	0.82	0.93	8.97
Spring	0.96	0.99	13.01
Summer	0.97	0.99	11.38
Autumn	0.98	0.96	10.77
***R***_***eco***_			
Winter	0.80	0.94	8.45
Spring	0.96	1.00	14.29
Summer	0.97	1.00	11.11
Autumn	0.99	0.97	10.35
***GPP***			
Winter	0.85	0.96	2.23
Spring	0.92	0.99	7.26
Summer	0.93	0.98	7.53
Autumn	0.96	0.99	2.85

Slope of regression model, R^2^: coefficient of determination, RMSE: root mean square error. Random Forest training using the data range from 2003 to 2007, testing using 2008.

**Fig. 11:**
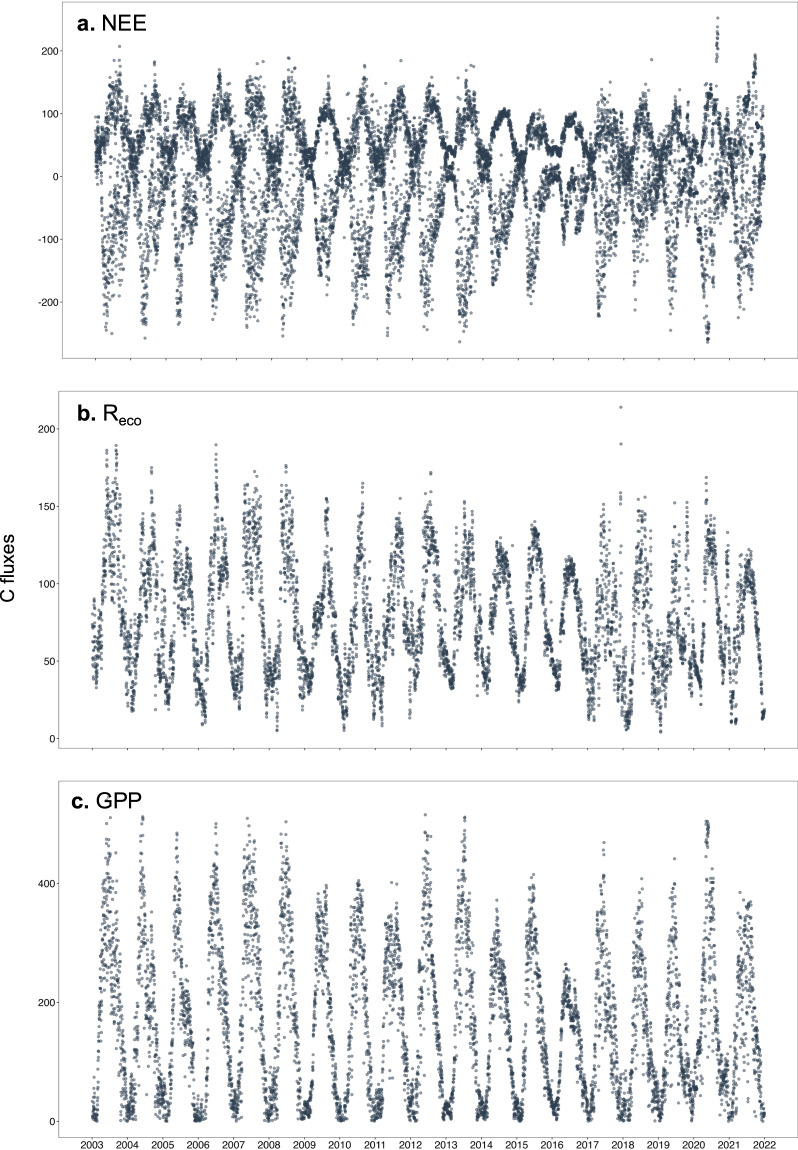
Daily C fluxes after gap-filling using the random forest models. (**a**) Daytime and nighttime NEE, (**b**) Daytime R_eco_, (**c**) Nighttime GPP.

Finally, after all steps of validation and sensitivity analysis, we used the RF models trained with 2003–2007 to gap-fill missing values in our EC time series. To verify their uncertainty, we obtained the standard deviation of important performance metrics (RMSE, MAE, and R^2^) after running the models 50 times (Table 6). The results of each model are presented in the Supplementary Table 2”. All models presented low uncertainty and the gap-filled values of C fluxes were obtained by averaging their outputs. Finally, visual screening was used to check whether the RF models were able to detect and reproduce the temporal component of the C fluxes (NEE, daytime GPP, and nighttime R_eco_) across the long-term time series. The imputed databases presented similar seasonality along the years, that is, with the highest C sequestration and respiration in the summer and spring (Fig. 11).

**Table 6:** Mean and standard deviation (SD) based on 50 random models for each response variable.

Metric	Mean	SD
***NEE***		
RMSE	29.56	0.0945
R^2^	0.88	0.0007
MAE	22.12	0.0870
***R***_***eco***_		
RMSE	21.50	0.0781
R^2^	0.92	0.0007
MAE	16.74	0.0670
***GPP***		
RMSE	29.49	0.0824
R^2^	0.95	0.0004
MAE	21.40	0.0605

RMSE: root mean square error, R^2^: coefficient of determination, MAE: mean absolute error. Random forest training using the data range from 2003 to 2007, testing using 2008.

## Usage Notes

Our datasets have been produced using best-practice processing and quality check procedures as recommended in the literature^2,8^. The dataset^3^ can be used stand-alone to address climate-flux relationships at both fine-scale (half-hour) and coarser (daily) temporal resolutions for this model ecosystem; it is of particular value for improved understanding of the mechanisms underlying variation in grassland production and C sequestration, as well as exploring the proximal and distal climatic drivers of single anomalous events^46^. The data can also be used to explore as part of a larger database to answer broader questions related to interactive effects of management and climate on grassland functions across pedoclimatic gradients, analyses of trade-offs and/or synergies between a wider range of ecosystem services and energy fluxes in the food-web^42^, or cross-ecosystem comparisons. Further, the RF pipeline for gap-filling described here can be transposed to other flux datasets, independent of temporal resolution, and used to facilitate the compilation of older datasets.

The half-hour dataset presents important variables, i.e., time stamp (YYYYMMDDHHMM), quality flags, and statistical analysis (hard flags), which will be useful for final users in filtering and aggregating the dataset according to their objectives. We also present the original NEE, R_eco_, and GPP (“_original”) values, as well as those ones gapfilled using the different uStar thresholds (“_U05”, “_U50” and “_U95”). More detailed information about the use of EC data at different temporal resolutions can be found in numerous scientific publications, as well as on FLUXNET website (https://fluxnet.org). Missing values in half-hour dataset are indicated with NA, and column name descriptions are provided in the associated metadata file.

This long-term EC time series fills an important information gap for grassland systems. It is of particular value for improved understanding of the mechanisms underlying variation in grassland production and C sequestration, as well as exploring the proximal and distal climatic drivers of single anomalous events^46^. Finally, we emphasise that the use of long-term C-flux measurements helps to understand possible adaptation of grassland ecosystems to future climate changes. By using different statistical models, such as path analysis^47^, that explore the causal relationship among the variables, and machine learning algorithms^12,48^ to forecast C-fluxes for future periods, we can contribute to the development of management strategies to meet high-C sequestration and climate mitigation goals.

## Supplementary information

Supplementary information

## Data Availability

The code for climate variability calculation, EC post-processing, random forest algorithm used for gap-filling can be obtained with the flux dataset^3^.
