# Supplementary material for: Eighteen years of upland grassland carbon flux data: reference datasets, processing, and gap-filling procedure
Source: Sci Data. 2023 May 23;10:311. doi: 10.1038/s41597-023-02221-z (PMC10205705; doi:10.1038/s41597-023-02221-z)
Supplement: Supplementary file 1 — Supplementary information [file 41597_2023_2221_MOESM1_ESM.pdf]

Supplementary information

Table of content

Table S1. Comparison of EdiRe and Eddypro® parametrization steps applied on raw-data pre-processing in the present (EddyPro) dataset and that of Klumpp et al. (2011) (EdiRe).

Table S2: Training metrics of 50 random models for of net ecosystem exchange (NEE), gross primary productivity (GPP), and ecosystem respiration (R_{eco}). RMSE: root mean square error, R^2: coefficient of determination, MAE: mean absolute error.

Table S1. Comparison of EdiRe and Eddypro® parametrization steps applied on raw-data pre-processing in the present (EddyPro) dataset and that of Klumpp et al. (2011) (EdiRe).

Pre-processing steps	EdiRe	EddyPro	Implications of EddyPro settings (where different)
		Processing options	
Determination of mean, SD, minimum, maximum values	Done	Done	
Coordinate rotation for tilt correction	Double rotation	Planar fit (Wilczak *et al.* 2001)	*Increase in processing details*, considers the sloping topography, when the mean vertical wind component or cross-stream stresses differs from zero.
Time lag compensation	Constant lag time	Automatic time lag optimization	*Increase in processing details,* determines the most suitable **nominal time lag** and plausibility window (Minimum and Maximum time lags)
Turbulent fluctuations, Detrend Method	Block average (Gash and Culf, 1996)	Same	
WPL correction (open-path)	Ibrom *et al.* (2007)[46]	Burba *et al.* (2008), Grelle and Burba (2007), Jarvi *et al.* (2009)	*Increase in processing details*
Correcting apparent off-season CO_2 uptake due to surface heating (LI-7500 only)	Not taken into account	Simple linear regression (Burba *et al.,* 2008; Grelle and Burba, 2007; Jarvi *et al.,* 2009)	*Increase in processing details,* accounts for the additional instrument-related sensible heat flux, related to instrument surface heating/cooling.
Quality check (0-1-2 system)	Mauder and Foken (2004)	Same	
Footprint estimation	Kljun *et al.* (2004) but estimated at post-processing	Kljun *et al.* (2004) but estimated at pre-processing	*Increase in precision,* accounts for field boundaries based on footprint size.

	Statistical tests for raw data screening		
Despiking	3 iterations of the procedure with 3; 3.3; and 3.63 standard deviation. 4 consecutive spikes are removed	20 iterations of the procedure with 3.5 standard deviation (CO_2 and H_2O; Vickers and Mahrt, 1997; Mauder, 2013), 3 consecutive spikes are removed	
Amplitude resolution	Vickers and Mahrt (1997)	Same	*Increase in processing details,* tests can be individually used after EddyPro processing according to clean half-hour dataset at post-processing step.
Drop-outs	Not taken into account	Vickers and Mahrt (1997)	
Absolute limits	Not taken into account	Vickers and Mahrt (1997)	
Skewness and Kurtosis	Vickers and Mahrt (1997)	Same	
Time lags	Vickers and Mahrt (1997)	Same	
Steadiness of horizontal wind	Not taken into account	Vickers and Mahrt (1997)	
Random uncertainty estimation	Not taken into account	Finkelstein and Sims (2001) with cross-correlation first crossing 1/e	

	Spectral analyses and corrections		
Spectra and co-spectra calculation	Power-of-two samples to speed up the FFT	Same	
QA/QC of spectra and co-spectra	Vickers and Mahrt (1997)	Same	
Filter (co)spectra according to micrometeorological quality test results	Low data quality (flag value = 2) Mauder and Foken (2006)	Same	
High-pass filtering correction	Moncrieff *et al.* (2004)	Same	
Low-pass filtering correction	Moncrieff *et al.* (1997)	Same	

Table S2. Training metrics of 50 random models for of net ecosystem exchange (NEE), gross primary productivity (GPP), and ecosystem respiration (R_{eco}). RMSE: root mean square error, R^2: coefficient of determination, MAE: mean absolute error.

	training: 2004-2008, testing: 2003			training: 2003-2007, testing: 2008		
**Models**	**RMSE**	**R2**	**MAE**	**RMSE**	**R2**	**MAE**
			Net ecosystem exchange (NEE)			
Model - 1	37	0.845	27.5	29.4	0.885	22
Model - 2	36.8	0.846	27.5	29.4	0.885	21.9
Model - 3	37.1	0.843	27.5	29.4	0.885	22
Model - 4	37	0.844	27.5	29.4	0.886	22
Model - 5	37	0.844	27.4	29.5	0.885	22.1
Model - 6	37	0.845	27.5	29.5	0.884	22.1
Model - 7	37	0.844	27.4	29.5	0.885	22
Model - 8	37	0.844	27.4	29.5	0.884	22.1
Model - 9	37.1	0.844	27.5	29.5	0.885	22
Model - 10	36.9	0.845	27.4	29.5	0.885	22
Model - 11	37.1	0.843	27.6	29.5	0.885	22
Model - 12	37.1	0.844	27.5	29.5	0.885	22
Model - 13	37.3	0.842	27.6	29.5	0.885	22.1
Model - 14	37	0.845	27.4	29.5	0.885	22.1
Model - 15	36.8	0.846	27.4	29.5	0.884	22.1
Model - 16	36.9	0.845	27.4	29.5	0.885	22.1
Model - 17	37	0.844	27.4	29.5	0.885	22.1
Model - 18	36.9	0.845	27.4	29.5	0.885	22.1
Model - 19	36.8	0.846	27.4	29.5	0.885	22.2
Model - 20	37	0.845	27.4	29.5	0.885	22.1
Model - 21	36.9	0.845	27.4	29.5	0.884	22.1
Model - 22	37.1	0.844	27.4	29.5	0.885	22.1
Model - 23	37.1	0.843	27.5	29.5	0.885	22.1
Model - 24	36.9	0.845	27.4	29.5	0.884	22.1
Model - 25	36.9	0.845	27.4	29.5	0.885	22.2
Model - 26	37	0.844	27.4	29.6	0.884	22.1
Model - 27	36.9	0.845	27.4	29.6	0.885	22.1
Model - 28	36.8	0.846	27.3	29.6	0.884	22.2
Model - 29	36.9	0.845	27.5	29.6	0.885	22.2
Model - 30	36.9	0.845	27.4	29.6	0.884	22.2
Model - 31	37.1	0.844	27.5	29.6	0.884	22.1
Model - 32	37	0.845	27.4	29.6	0.884	22.1
Model - 33	37.1	0.843	27.5	29.6	0.884	22.1
Model - 34	37.1	0.843	27.6	29.6	0.885	22.2
Model - 35	37	0.844	27.4	29.6	0.884	22.2
Model - 36	36.7	0.846	27.2	29.6	0.884	22.1
Model - 37	36.9	0.845	27.4	29.6	0.884	22.2
Model - 38	36.9	0.845	27.4	29.6	0.884	22.2
Model - 39	37.1	0.844	27.6	29.6	0.885	22.2

Model - 40	37.1	0.844	27.5	29.6	0.884	22.1
Model - 41	37	0.845	27.5	29.6	0.884	22.2
Model - 42	36.8	0.846	27.3	29.6	0.885	22.1
Model - 43	37	0.845	27.4	29.7	0.883	22.2
Model - 44	36.8	0.846	27.3	29.7	0.883	22.3
Model - 45	36.8	0.846	27.3	29.7	0.884	22.2
Model - 46	36.9	0.845	27.3	29.7	0.884	22.2
Model - 47	36.8	0.846	27.4	29.7	0.883	22.2
Model - 48	36.8	0.846	27.3	29.7	0.884	22.2
Model - 49	37	0.844	27.5	29.8	0.883	22.3
Model - 50	37	0.844	27.5	29.8	0.883	22.3

Ecosystem respiration (Reco)

Model - 1	29.6	0.903	21.7	21.4	0.917	16.7
Model - 2	29.6	0.904	21.6	21.4	0.917	16.6
Model - 3	29.7	0.903	21.7	21.6	0.915	16.8
Model - 4	29.4	0.905	21.5	21.5	0.916	16.8
Model - 5	29.8	0.902	21.8	21.5	0.916	16.7
Model - 6	29.6	0.905	21.6	21.6	0.916	16.8
Model - 7	29.7	0.904	21.7	21.6	0.915	16.8
Model - 8	29.6	0.903	21.6	21.4	0.917	16.7
Model - 9	29.6	0.904	21.5	21.4	0.916	16.7
Model - 10	29.6	0.903	21.6	21.6	0.916	16.8
Model - 11	29.9	0.903	21.8	21.5	0.916	16.7
Model - 12	29.6	0.903	21.6	21.6	0.915	16.8
Model - 13	29.7	0.903	21.7	21.5	0.916	16.7
Model - 14	29.5	0.905	21.5	21.5	0.916	16.7
Model - 15	29.7	0.904	21.7	21.4	0.917	16.7
Model - 16	29.5	0.905	21.5	21.5	0.916	16.7
Model - 17	29.6	0.903	21.7	21.4	0.917	16.6
Model - 18	29.6	0.904	21.5	21.6	0.915	16.8
Model - 19	29.7	0.902	21.7	21.5	0.916	16.8
Model - 20	29.7	0.904	21.6	21.6	0.915	16.8
Model - 21	29.7	0.903	21.6	21.4	0.917	16.7
Model - 22	29.7	0.902	21.7	21.5	0.916	16.8
Model - 23	29.5	0.906	21.7	21.4	0.917	16.7
Model - 24	29.6	0.902	21.6	21.6	0.916	16.8
Model - 25	29.8	0.902	21.7	21.5	0.916	16.8
Model - 26	29.6	0.903	21.6	21.6	0.916	16.8
Model - 27	29.8	0.903	21.8	21.5	0.916	16.8
Model - 28	29.6	0.904	21.7	21.4	0.917	16.7
Model - 29	29.6	0.904	21.6	21.6	0.915	16.8
Model - 30	29.5	0.902	21.5	21.5	0.916	16.7
Model - 31	29.6	0.903	21.6	21.5	0.916	16.7
Model - 32	29.4	0.904	21.5	21.6	0.915	16.8
Model - 33	29.7	0.903	21.7	21.4	0.916	16.7
Model - 34	29.5	0.903	21.6	21.5	0.917	16.7
Model - 35	29.4	0.905	21.6	21.5	0.916	16.8

Model - 36	29.6	0.903	21.6	21.4	0.917	16.7
Model - 37	29.5	0.903	21.5	21.6	0.916	16.8
Model - 38	29.6	0.905	21.6	21.4	0.917	16.7
Model - 39	29.8	0.904	21.7	21.4	0.917	16.6
Model - 40	29.6	0.904	21.6	21.4	0.917	16.7
Model - 41	29.6	0.904	21.6	21.6	0.916	16.8
Model - 42	29.6	0.904	21.6	21.6	0.916	16.8
Model - 43	29.5	0.905	21.6	21.5	0.916	16.7
Model - 44	29.6	0.904	21.7	21.6	0.915	16.9
Model - 45	29.6	0.903	21.7	21.5	0.917	16.6
Model - 46	29.7	0.903	21.7	21.5	0.916	16.8
Model - 47	29.7	0.903	21.6	21.6	0.915	16.8
Model - 48	29.7	0.903	21.6	21.5	0.916	16.7
Model - 49	29.4	0.904	21.5	21.5	0.916	16.7
Model - 50	29.7		21.8	21.5	0.916	16.7

Gross primary productivity (GPP)

Model - 1	43.1	0.913	29.1	29.5	0.952	21.4
Model - 2	43.2	0.911	29.2	29.5	0.952	21.4
Model - 3	42.9	0.913	29	29.5	0.952	21.5
Model - 4	43	0.912	29.1	29.5	0.952	21.5
Model - 5	42.8	0.913	28.9	29.5	0.952	21.4
Model - 6	42.7	0.913	28.9	29.3	0.952	21.3
Model - 7	42.9	0.913	28.9	29.5	0.952	21.4
Model - 8	42.9	0.912	29	29.5	0.952	21.4
Model - 9	42.8	0.913	28.9	29.7	0.951	21.5
Model - 10	42.9	0.912	29	29.4	0.952	21.4
Model - 11	42.9	0.913	29	29.4	0.952	21.3
Model - 12	42.8	0.913	29	29.6	0.951	21.4
Model - 13	42.8	0.913	28.9	29.5	0.952	21.4
Model - 14	42.9	0.913	29	29.6	0.951	21.4
Model - 15	42.9	0.913	29	29.5	0.952	21.4
Model - 16	43	0.912	29	29.5	0.952	21.4
Model - 17	42.8	0.913	29	29.4	0.952	21.4
Model - 18	42.7	0.913	29	29.5	0.952	21.4
Model - 19	42.8	0.913	28.9	29.5	0.952	21.4
Model - 20	42.7	0.913	28.9	29.4	0.952	21.3
Model - 21	42.9	0.912	29	29.6	0.952	21.5
Model - 22	42.9	0.912	29.1	29.6	0.951	21.4
Model - 23	42.8	0.912	29	29.4	0.952	21.3
Model - 24	42.8	0.913	28.9	29.4	0.952	21.4
Model - 25	43	0.912	29.1	29.6	0.951	21.5
Model - 26	42.7	0.913	29	29.4	0.952	21.3
Model - 27	42.8	0.913	29	29.5	0.952	21.4
Model - 28	43	0.912	29.1	29.4	0.952	21.3
Model - 29	42.8	0.913	28.9	29.3	0.952	21.3
Model - 30	43	0.913	29.1	29.5	0.952	21.4
Model - 31	42.7	0.913	28.9	29.5	0.952	21.4

Model - 32	42.9	0.913	29.1	29.5	0.952	21.4
Model - 33	42.9	0.912	29	29.4	0.952	21.4
Model - 34	43	0.913	29.1	29.5	0.952	21.4
Model - 35	43.1	0.912	29.1	29.5	0.952	21.5
Model - 36	42.7	0.913	28.8	29.5	0.952	21.3
Model - 37	42.8	0.913	29	29.5	0.952	21.4
Model - 38	42.7	0.913	28.9	29.5	0.952	21.4
Model - 39	42.9	0.913	29	29.6	0.951	21.4
Model - 40	42.9	0.913	29	29.4	0.952	21.4
Model - 41	42.8	0.913	29	29.6	0.951	21.4
Model - 42	42.8	0.912	28.9	29.5	0.951	21.3
Model - 43	43	0.912	29.1	29.4	0.952	21.4
Model - 44	42.8	0.913	29	29.5	0.952	21.4
Model - 45	42.9	0.912	29.1	29.4	0.952	21.3
Model - 46	42.9	0.912	29	29.5	0.952	21.5
Model - 47	43	0.912	29.1	29.6	0.951	21.5
Model - 48	42.9	0.913	29.1	29.6	0.952	21.4
Model - 49	42.9	0.912	29	29.4	0.952	21.4
Model - 50	42.6	0.913	28.8	29.5	0.951	21.4
